# Lipidomics Analysis of Behavioral Variant Frontotemporal Dementia: A Scope for Biomarker Development

**DOI:** 10.3389/fneur.2018.00104

**Published:** 2018-02-28

**Authors:** Woojin Scott Kim, Eve Jary, Russell Pickford, Ying He, Rebekah M. Ahmed, Olivier Piguet, John R. Hodges, Glenda M. Halliday

**Affiliations:** ^1^Brain and Mind Centre, Sydney Medical School, The University of Sydney, Sydney, NSW, Australia; ^2^Neuroscience Research Australia, Sydney, NSW, Australia; ^3^School of Medical Sciences, University of New South Wales, Sydney, NSW, Australia; ^4^Bioanalytical Mass Spectrometry Facility, University of New South Wales, Sydney, NSW, Australia; ^5^ARC Centre of Excellence in Cognition and Its Disorders, Sydney, NSW, Australia; ^6^School of Psychology, The University of Sydney, Sydney, NSW, Australia

**Keywords:** frontotemporal dementia, lipids, dyslipidemia, lipidomics, Alzheimer’s disease, biomarker, hypertriglyceridemia

## Abstract

Behavioral variant frontotemporal dementia (bvFTD) is the most prevalent form of FTD syndromes. bvFTD is characterized clinically by changes in behavior and cognition and pathologically by focal brain atrophy and concomitant loss of lipids. bvFTD is further characterized by eating abnormalities that result in dyslipidemia. Although dyslipidemia is apparent in bvFTD, very little is known about global lipid changes in bvFTD and lipid dysregulation underlying bvFTD. Here, we undertook a comprehensive lipidomics analysis of blood plasma from patients with bvFTD, patients with Alzheimer’s disease (AD) and controls, using liquid chromatography-tandem mass spectrometry, with the aim of understanding lipid dysregulation in bvFTD. In our analysis, we detected all four major classes of lipids (glycerolipids, phospholipids, sphingolipids, sterols), 17 subclasses of lipids, and 3,225 putative individual lipid species in total, as well as a group of dietary lipids. We found that the levels of numerous lipid species were significantly altered in bvFTD compared to AD and control. We found that the total abundance of triglyceride (TG) increased significantly in bvFTD, whereas phosphatidylserine and phosphatidylglycerol decreased significantly in bvFTD. These results suggest manifestation of hypertriglyceridemia and hypoalphalipoproteinemia in bvFTD. We also identified five lipid molecules—TG (16:0/16:0/16:0), diglyceride (18:1/22:0), phosphatidylcholine (32:0), phosphatidylserine (41:5), and sphingomyelin (36:4)—that could potentially be used for developing biomarkers for bvFTD. Furthermore, an analysis of plant lipids revealed significant decreases in monogalactosyldiacylglycerol and sitosteryl ester in bvFTD, indicating altered eating behavior in bvFTD. This study represents the first lipidomics analysis of bvFTD and has provided new insights into an unrecognized perturbed pathology in bvFTD, providing evidence in support of considerable lipid dysregulation in bvFTD.

## Introduction

Frontotemporal dementia syndromes are a common cause of early onset (<65 years of age) dementia (1). These syndromes are characterized by dramatic changes in personality, language, behavior, and social conduct. The most prevalent syndrome is behavioral variant frontotemporal dementia (bvFTD) (2); less prevalent are a number of primary progressive aphasias (3). bvFTD patients are commonly misdiagnosed as Alzheimer’s disease (AD) because of overlap in clinical presentations, although the regions of the brain affected and the types of underlying cellular pathologies differ in the two disorders. Recent advances in the genetics of frontotemporal dementia syndromes have identified aberration in three key genes, *C9ORF72* (4, 5), *progranulin* (6), and *tau* (7). A genetic biomarker for the *C9ORF72* gene expansion has been developed, and genetic screening of bvFTD for this expansion is now commonly performed at an early stage of diagnosis. Increasing effort is being made to develop blood biomarkers based on gene products of *progranulin* and *tau* with the aim of differentiating the underlying brain proteinopathies in bvFTD, as well as for target validation in clinical trials (8, 9).

One aspect of the neuropathology of bvFTD that has not been exploited in the development of selective biomarkers are the changes in lipids. In contrast to AD, there is considerable early loss of significant amounts of brain tissue in all frontotemporal dementia syndromes with concomitant loss of lipids (10–13). Lipids are a major constituent of brain tissue, making up 39.6% of gray matter and 64.6% of white matter (14). Another aspect of the neuropathology of bvFTD that contributes toward altered lipid status is the behavioral changes that impact on diet and eating behaviors. Key features of bvFTD are excessive or binge eating, increased consumption of sweet foods (sugar) and alcohol, and increased body mass index (BMI) (15–17). In a recent study, blood lipid analyses showed increased levels of triglyceride (TG) and decreased levels of HDL-cholesterol in a cohort of bvFTD compared to controls, indicative of lipid metabolic abnormality (18). Apart from total TG and total cholesterol, no other lipids have been measured in these syndromes; no individual lipid species have been measured.

Here, we undertook a comprehensive analysis of global plasma lipid levels in bvFTD compared with AD and controls using untargeted lipidomics technology. Lipidomics, based on HPLC and mass spectrometry, allows detection and quantification of individual molecular species of a broad range of lipids. It has been extensively used in biomedical research and has provided invaluable data in understanding the pathogenesis of a number of diseases. Lipidomics has also provided the scope for use in disease classification and biomarker development. Thus far, no lipidomics analysis has been reported for bvFTD. The primary aim of our study was to generate profiles of lipids present in blood plasma from patients with bvFTD and to determine any changes compared to patients with AD and controls without neurological or psychiatric disorders. The secondary aim was to identify individual lipid species that could be used in follow-up studies to develop potential biomarkers to objectively distinguish bvFTD patients from AD patients and controls.

## Materials and Methods

### Chemicals and Materials

Lipids were extracted using chloroform, methanol, and isopropanol (Sigma Aldrich, St. Louis, MO, USA) and ultrapure water (Millipore). All solvents used were HPLC grade or higher. Glass pipettes and tubes were used wherever possible, and the use of plasticware was minimized during lipid extraction to avoid contamination of samples. Glass tubes and glass transfer pipettes were purchased from Sigma and vWR. Lipid internal standards (ISTDs) were purchased from Avanti Polar Lipids Inc. (Alabaster, AL, USA). These include phosphatidylcholine (PC, 19:0), sphingomyelin (SM, 12:0), phosphatidylethanolamine (PE, 17:0), phosphatidylglycerol (PG, 17:0), phosphatidylserine (PS, 17:0), phosphatidic acid (PA, 17:0), ceramide (Cer, d18:1, 12:0), diglyceride (DG, 1,3 18:0 d5), cholesteryl ester (ChE, 19:0), monoglyceride (MG, 17:0), TG mix d5 (Avanti Code LM-6000), DG mix d5 (Avanti Code LM-6001), phosphatidylinositol (PI, 17:0 14:1), C12 GluCer, C12 sulfatide, C17 Cer, C17 sphingosine (So), C17 S1P, C12 C1P, D3 C20 fatty acid, and C12 LacCer. Lipid ISTDs were prepared as a mixture at 10 pmol/μl in methyl-tert butyl ether and methanol (MTBE:methanol, 1:1 v/v).

### Patient Blood Collection

Patients with bvFTD (M/F, 11/5), patients with AD (M/F, 8/6) and healthy controls (M/F, 8/14) were included in the study. The mean age of these three groups were 65.0 ± 6.5, 70.9 ± 5.7, and 73.7 ± 5.4 years, respectively. They were recruited from FRONTIER, the frontotemporal dementia clinical research group at Neuroscience Research Australia, Sydney. All patients underwent a comprehensive neurologic and cognitive assessment and their clinical status established as previously described (18), and they met their respective current clinical diagnostic criteria (16, 19). The controls were recruited from a panel of healthy study volunteers (18) and had no neurological (i.e., no evidence of cognitive impairment) or psychiatric disorders or family histories of such disorders. Fasted blood was collected, and plasma was prepared by centrifugation at 3,500 rpm for 10 min at 4°C, which was then aliquoted and stored at −80°C until use. This study was approved by the University of New South Wales human ethics committee (approval number: HC12573), and the blood was obtained following written informed consent from the participant and/or primary carer.

### Lipid Extraction

Plasma was thawed on ice prior to extraction. An aliquot of 80 µl of plasma was mixed with 10 µl of the internal standard in a glass tube. Lipid extraction was based on the method by Bligh and Dyer (20). Briefly, methanol (600 µl), chloroform (1,000 µl), and ultrapure water (500 µl) were added sequentially and vortexed each time. Samples were then centrifuged at 3,000 rpm for 25 min at room temperature. The lower (solvent) phase was collected and transferred into a new glass tube using a glass Pasteur pipette. Chloroform (600 µl) was added, vortexed, and centrifuged at 3,000 rpm for 25 min. The lower phase was collected and transferred into a new glass tube and dried under nitrogen gas. The dried lipid samples were reconstituted in 100 µl of isopropanol/methanol (1:1).

### LC-MS/MS Protocol

Lipid extract (10 µl) was analyzed using a Q-Exactive Plus Mass Spectrometer coupled to a U3000 UPLC system (ThermoFisher Scientific). Chromatography was performed at 60°C on a Waters CSH C18 UHPLC column 2.1 × 100 mm, 1.8 µM with VanGuard guard column.

Solvent A was 6:4 acetonitrile:water and Solvent B was 1:9 acetonitrile:isopropanol, both with 10 mM ammonium formate and 0.1% formic acid. Lipids were chromatographed according to the method of Castro-Perez et al (21). Briefly, a 30-min gradient running from 30 to 100% of solvent B was performed, eluting lipids in order of hydrophobicity. Column eluate was directed into the electrospray ionization (ESI) source of the mass spectrometer where a HESI probe was employed. Source parameters were broadly optimized on a range of lipid standards prior to the analysis. The mass spectrometer was run in data-dependent acquisition mode. A survey scan over the mass range 200–1,200 at resolution 70 K was followed by 10 data dependent MS/MS scans on the most intense ions in the survey at 15 K resolution. Dynamic exclusion was used to improve the number of ions targeted. Cycle time was approximately 1 s. Samples were run in both positive and negative polarities. The samples were run in a random order (generated using Microsoft Excel). This is important to avoid batch effects/changing instrument performance effects. Data were analyzed in LipidSearch software 4.1.16. Data were searched against the standard Lipidsearch database with all common mammalian lipid classes included. The search results were then grouped according to sample type and aligned for differential analysis. Aligned data (containing lipid identity, retention time, peak area, etc.) were exported to Excel software. Relative abundance of lipids was obtained from peak areas normalized to ISTDs. Individual species that were significantly altered in bvFTD and AD compared to control were presented as fold changes on linear scale. Fold changes were calculated by dividing disease abundance by control abundance and then subtracting by 1; zero, therefore, represents no change. Increased species are represented by red symbols above the zero line (e.g., 0.5 = 50% increase) and decreased species are represented by blue symbols below the zero line (e.g., −0.5 = 50% decrease).

### Statistics

Statistical analyses were performed using SPSS Statistics software (IBM, Chicago, IL, USA). Multivariate analyses (general linear model) covarying for age and gender were used to determine differences in lipid levels in the control, bvFTD, and AD clinical groups with *post hoc* statistical significance set at *p* < 0.05. Pearson correlations were used to determine if changes in the level of a lipid were associated with all other lipids with statistical significance set at *p* < 0.05.

## Results

### A Global Analysis of bvFTD Plasma Lipids

Behavioral variant frontotemporal dementia is characterized by brain atrophy and concomitant loss of lipids. bvFTD is further characterized by eating abnormalities and changes to BMI and metabolism. However, very little is known about changes in the level and distribution of blood lipids in bvFTD. Here, we undertook a comprehensive lipidomics analysis of blood plasma from patients with bvFTD, AD, and controls without dementia with the potential aim of developing lipid biomarkers for bvFTD. We measured all major classes of lipids using liquid chromatography-tandem mass spectrometry. The total relative abundance (normalized to ISTDs) of each lipid class in each cohort is shown in Table 1. Changes to lipid levels in bvFTD and AD were analyzed using a single statistical approach—multivariate tests covarying for age and gender, and each lipid class is described in detail below. Both age (*p* = 0.368) and gender (*p* = 0.176) had no significant effect on lipid levels. The number of species identified in each lipid class is also shown in Table 1 and described in detail below. We also identified individual lipid species that could potentially be used for developing biomarkers for bvFTD that differentiate them from both AD patients and controls, and these are also described in detail below.

**Table 1:** Total relative abundance and number of species identified for each lipid class.

Lipid	Symbol	Species	Total abundance			SE			Significance		
			Con	Behavioral variant frontotemporal dementia (bvFTD)	Alzheimer’s disease (AD)	Con	bvFTD	AD	Con v bvFTD	Con v AD	bvFTD v AD
**Glycerolipids**											
Triglyceride	TG	1,115	28,288,797,070	37,809,429,330	35,132,579,390	2,315,247,310	2,834,542,577	2,651,164,228	0.0215*	0.0565	0.4973
Diglyceride	DG	282	798,173,211	1,150,203,620	1,088,099,179	115,173,752	141,006,493	131,884,197	0.0835	0.1025	0.7511
Monoglyceride	MG	22	15,367,133	19,978,654	17,477,209	1,851,801	2,267,148	2,120,477	0.1563	0.4547	0.4282

**Phospholipids**											
Phosphatidylcholine	PC	1,062	68,308,624,220	61,637,766,990	68,242,851,540	2,241,151,424	2,743,827,454	2,566,317,844	0.0916	0.9846	0.0878
Phosphatidylserine	PS	91	1,713,312,131	1,360,454,204	1,747,110,899	81,147,600	99,348,491	92,921,224	0.0154*	0.7841	0.0070*
phosphatidylethanolamine	PE	152	1,690,377,391	1,604,351,098	1,816,156,828	93,113,124	113,997,806	106,622,814	0.5955	0.3760	0.1848
Phosphatidylinositol	PI	29	110,809,533	85,315,521	115,238,810	10,804,479	13,227,855	12,372,090	0.1787	0.7874	0.1080
Phosphatidylglycerol	PG	21	352,956,480	188,169,159	272,377,988	41,974,952	51,389,667	48,065,055	0.0278*	0.2103	0.2412
Phosphatidic acid	PA	6	276,188,531	245,437,646	277,454,089	14,785,570	18,101,879	16,930,793	0.2349	0.9551	0.2065

**Sphingolipids**											
Ceramide	Cer	59	700,586,282	627,391,708	711,568,776	54,061,427	66,187,062	61,905,145	0.4374	0.8937	0.3617
Monoglycosylceramide	CerG1	34	306,532,770	296,609,130	303,378,080	25,956,537	31,778,423	29,722,545	0.8259	0.9363	0.8780
Ceramide phosphate	CerP	2	705,982	689,733	763,499	166,052	203,297	190,145	0.9551	0.8197	0.7938
Sphingomyelin	SM	229	17,238,766,380	16,558,095,510	18,595,049,360	828,842,335	1,014,746,407	949,098,241	0.6369	0.2845	0.1526
Sphingosine	So	3	36,750,547	32,289,386	39,606,196	2,424,937	2,968,835	2,776,768	0.2926	0.4398	0.0807
Sphingosine phosphate	Sop	2	561,125	420,566	537,341	91,932	112,551	105,270	0.3809	0.8648	0.4561

**Sterols**											
Cholesteryl ester	ChE	44	2,036,113,428	1,720,063,142	1,851,966,097	104,534,543	127,980,977	119,701,356	0.0868	0.2497	0.4591
Zymosteryl ester	ZyE	22	945,498,557	832,938,261	862,188,966	45,898,483	56,193,222	52,557,848	0.0868	0.2497	0.4591

**Dietary lipids**											
Monogalactosyldiacylglycerol	MGDG	12	461,200,788	391,370,141	485,257,104	18,164,178	22,238,288	20,799,601	0.0310*	0.3854	0.0037*
Stigmasteryl ester	StE	3	505,584,209	465,560,451	449,560,609	29,211,953	35,764,008	33,450,286	0.4320	0.2107	0.7473
Sitosteryl ester	SiE	7	42,637,336	28,208,118	34,262,783	2,733,091	3,346,106	3,129,632	0.0037*	0.0483*	0.1964
Wax ester	WE	28	902,221,864	789,333,910	800,780,727	37,356,450	45,735,264	42,776,459	0.0870	0.0788	0.8569

Multivariate analyses covarying for age and gender were used to determine differences in lipid levels between the groups; *p < 0.05.

### Analysis of Glycerolipids

**Figure 1:**
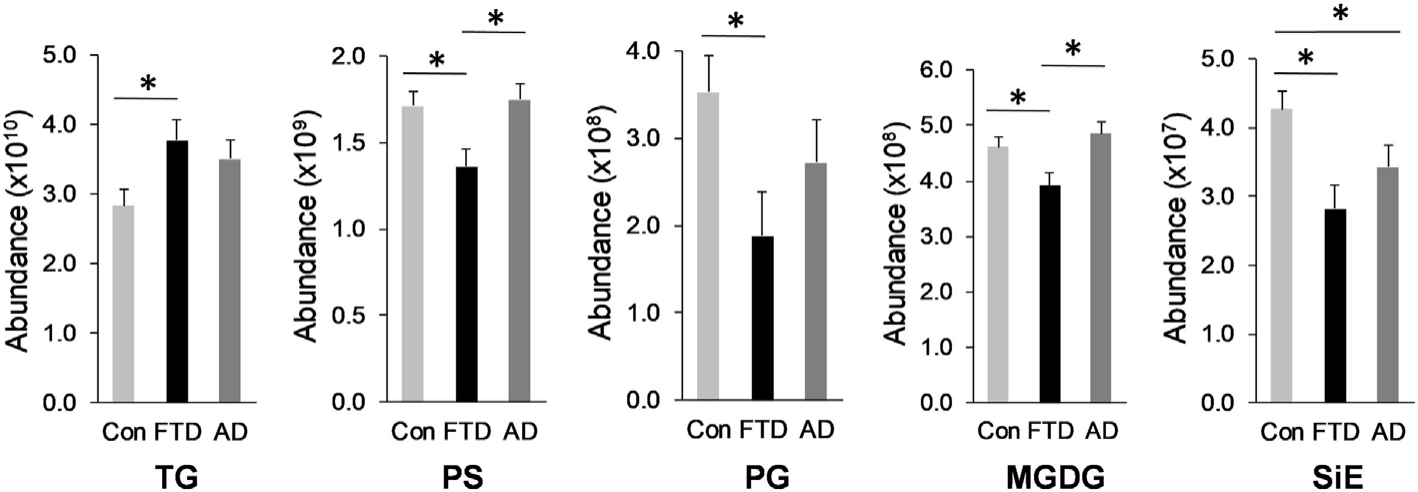
Lipids that were significantly altered in behavioral variant frontotemporal dementia. Total relative abundance of lipids following multivariate analyses covarying for age and gender; Data represent mean and SE as error bars, *p < 0.05. TG, triglyceride; PS, phosphatidylserine; PG, phosphatidylglycerol; MGDG, monogalactosyldiacylglycerol; SiE, sitosteryl ester.

Glycerolipid is a class of lipids that account for a high proportion of total lipids in plasma. Structurally glycerolipids contain a glycerol backbone linked to one, two, or three fatty acid chains. The main subclass of glycerolipids in plasma is TG also called triacylglycerol. TG is normally transported in the blood as a constituent of lipoproteins. The two other subclasses in this class are precursors of TG; they are DG and MG. Both are also present in lipoproteins. We analyzed all three subclasses in our cohorts and as expected TG was the most abundant of the three subclasses (Table 1). TG also had the highest number of lipid species with 1,115 species identified (Table 1). TG was significantly increased in bvFTD compared to control (Figure 1). There was a non-significant increase (*p* = 0.084) in DG in bvFTD compared to control with no significant change in MG.

**Figure 2:**
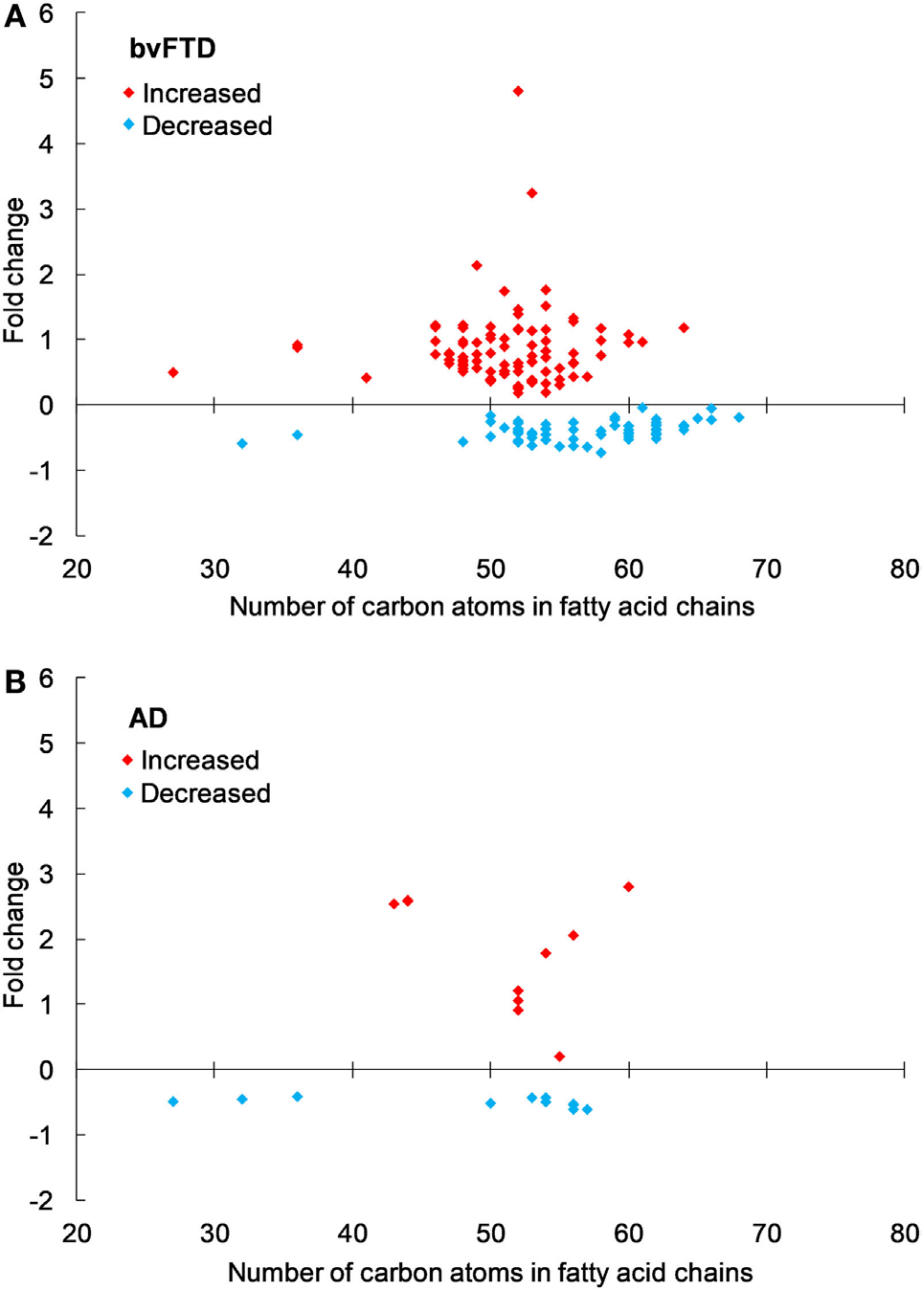
Triglyceride species that were significantly altered in **(A)** behavioral variant frontotemporal dementia (bvFTD) and **(B)** Alzheimer’s disease (AD) compared to control. Each symbol represents a lipid species with different number of carbon atoms in fatty acid chains; red symbols are those that are significantly increased and blue symbols significantly decreased; *p* < 0.05. The data presented are fold changes on linear scale. Zero represents no change; 0.5 fold change represents 50% increase; and −0.5 represents 50% decrease.

An analysis of each individual species showed that 97 TG species were significantly increased in bvFTD compared to control (Figure 2A; Table 2), whereas only 10 TG species were significantly increased in AD compared to control (Figure 2B; Table 2). There were also TG species that decreased significantly in bvFTD, but fewer in number than those that increased significantly (Figure 2A; Table 2). A few of the species overlapped between bvFTD and AD. Furthermore, the mean fold change of these species that increased significantly in bvFTD was 0.84, whereas the mean fold change of these species that decreased significantly in FTD was only 0.39. We identified two glycerolipid species that could be potentially used for developing biomarkers for differentiating bvFTD from AD and controls (Table 3). One is a TG species (CalcMz = 824.77) with 16 carbons in each of the three fatty acid chains, and the other is a DG species (CalcMz = 696.65) with 18 and 22 carbons in each of the two fatty acid chains.

**Table 2:** Individual triglyceride species that were significantly altered (*p* < 0.05) in behavioral variant frontotemporal dementia (bvFTD) and Alzheimer’s disease (AD) compared to control.

Species	CalcMz	C atoms	Fold change	Species	CalcMz	C atoms	Fold change	Species	CalcMz	C atoms	Fold change
**bvFTD**											

TG(27:1)	528.4259	27	0.4982	TG(52:1)	878.8171	52	0.2841	TG(55:3)	916.8328	55	0.5622
TG(32:0)	600.5198	32	−0.5899	TG(52:1)	878.8171	52	0.5901	TG(55:4)	914.8171	55	0.3923
TG(36:0)	656.5824	36	0.8793	TG(52:1)	878.8171	52	1.4626	TG(55:6)	910.7858	55	0.3078
TG(36:0)	656.5824	36	−0.4573	TG(52:1)	878.8171	52	1.1662	TG(55:7)	908.7702	55	−0.6356
TG(36:0)	640.5875	36	0.9205	TG(52:2)	876.8015	52	−0.3672	TG(56:1)	934.8797	56	1.3298
TG(41:6)	714.5667	41	0.4153	TG(52:2)	876.8015	52	−0.4142	TG(56:12)	895.6810	56	−0.3768
TG(46:1)	794.7232	46	0.7745	TG(52:2)	876.8015	52	−0.3657	TG(56:3)	930.8484	56	−0.2662
TG(46:1)	794.7232	46	1.2207	TG(52:2)	876.8015	52	−0.4340	TG(56:3)	930.8484	56	0.4327
TG(46:2)	792.7076	46	0.9764	TG(52:2)	876.8015	52	−0.5441	TG(56:3)	930.8484	56	0.6351
TG(46:3)	773.6654	46	1.1936	TG(52:2)	876.8015	52	−0.3999	TG(56:3)	930.8484	56	0.6489
TG(47:1)	808.7389	47	0.7937	TG(52:2)	876.8015	52	−0.2465	TG(56:4)	911.8062	56	1.2795
TG(47:1)	808.7389	47	0.7754	TG(52:2)	876.8015	52	0.1849	TG(56:5)	926.8171	56	−0.6279
TG(47:1)	808.7389	47	0.6329	TG(52:2)	876.8015	52	0.2489	TG(56:6)	924.8015	56	−0.5215
TG(47:2)	806.7232	47	0.6920	TG(52:2)	876.8015	52	−0.5704	TG(56:6)	924.8015	56	0.7912
TG(48:0)	824.7702	48	0.6871	TG(52:3)	874.7858	52	−0.2792	TG(57:2)	946.8797	57	0.4328
TG(48:1)	822.7545	48	0.5545	TG(52:3)	874.7858	52	0.2654	TG(57:8)	934.7858	57	−0.6421
TG(48:1)	822.7545	48	0.5114	TG(52:3)	874.7858	52	0.2886	TG(58:1)	962.9110	58	0.9877
TG(48:1)	822.7545	48	1.2236	TG(52:3)	874.7858	52	−0.5628	TG(58:12)	940.7389	58	−0.3962
TG(48:1)	822.7545	48	1.1809	TG(52:3)	874.7858	52	0.6426	TG(58:2)	960.8954	58	1.1730
TG(48:1)	822.7545	48	−0.5632	TG(52:3)	874.7858	52	1.3901	TG(58:2)	960.8954	58	0.7577
TG(48:2)	820.7389	48	0.7358	TG(52:4)	855.7436	52	4.8063	TG(58:7)	950.8171	58	−0.7300
TG(48:2)	820.7389	48	0.6278	TG(52:5)	870.7545	52	1.1495	TG(58:9)	946.7858	58	−0.4555
TG(48:3)	818.7232	48	0.9364	TG(52:7)	849.6967	52	0.2657	TG(59:10)	958.7858	59	−0.3153
TG(48:4)	799.6810	48	0.6024	TG(53:1)	892.8328	53	0.7524	TG(59:11)	956.7702	59	−0.1824
TG(48:5)	797.6654	48	0.6661	TG(53:2)	890.8171	53	0.9150	TG(59:11)	956.7702	59	−0.2204
TG(48:6)	795.6497	48	0.9740	TG(53:2)	890.8171	53	0.3567	TG(60:1)	990.9423	60	1.0776
TG(49:1)	836.7702	49	0.9555	TG(53:2)	890.8171	53	1.1339	TG(60:10)	972.8015	60	−0.3195
TG(49:2)	834.7545	49	0.5649	TG(53:2)	890.8171	53	−0.4249	TG(60:10)	972.8015	60	−0.4240
TG(49:2)	834.7545	49	0.6702	TG(53:3)	888.8015	53	0.6572	TG(60:11)	970.7858	60	−0.4395
TG(49:3)	832.7389	49	0.7762	TG(53:3)	888.8015	53	0.6660	TG(60:11)	970.7858	60	−0.3806
TG(49:6)	809.6654	49	2.1358	TG(53:3)	888.8015	53	0.3449	TG(60:11)	970.7858	60	−0.4818
TG(50:1)	850.7858	50	−0.2550	TG(53:3)	888.8015	53	3.2448	TG(60:13)	949.7280	60	−0.5261
TG(50:1)	850.7858	50	0.3641	TG(53:4)	869.7593	53	1.1373	TG(60:14)	947.7123	60	−0.4243
TG(50:1)	850.7858	50	0.5101	TG(53:5)	884.7702	53	−0.5024	TG(60:2)	988.9267	60	0.9567
TG(50:2)	848.7702	50	−0.1614	TG(53:5)	867.7436	53	0.3842	TG(61:12)	982.7858	61	−0.0408
TG(50:2)	848.7702	50	−0.4823	TG(53:6)	865.7280	53	0.3688	TG(61:2)	1002.9423	61	0.9656
TG(50:2)	848.7702	50	1.0191	TG(53:7)	880.7389	53	−0.6213	TG(62:11)	998.8171	62	−0.3718
TG(50:2)	848.7702	50	0.4075	TG(53:8)	878.7232	53	−0.4478	TG(62:11)	998.8171	62	−0.2155
TG(50:2)	848.7702	50	0.3828	TG(54:1)	906.8484	54	0.9792	TG(62:12)	996.8015	62	−0.5144
TG(50:2)	848.7702	50	1.0730	TG(54:2)	904.8328	54	−0.3648	TG(62:12)	996.8015	62	−0.4528
TG(50:2)	848.7702	50	1.1979	TG(54:2)	904.8328	54	−0.4537	TG(62:12)	996.8015	62	−0.3850
TG(50:3)	846.7545	50	0.7931	TG(54:2)	904.8328	54	−0.3722	TG(62:13)	994.7858	62	−0.3107
TG(50:3)	846.7545	50	0.3762	TG(54:3)	902.8171	54	−0.2946	TG(62:13)	994.7858	62	−0.2671
TG(50:6)	823.6810	50	0.3960	TG(54:3)	902.8171	54	0.1926	TG(62:13)	994.7858	62	−0.3061
TG(51:1)	864.8015	51	1.0157	TG(54:3)	902.8171	54	0.5077	TG(62:15)	973.7280	62	−0.4308
TG(51:1)	864.8015	51	1.7421	TG(54:3)	902.8171	54	1.5160	TG(64:12)	1024.8328	64	−0.3808
TG(51:2)	862.7858	51	0.8935	TG(54:3)	902.8171	54	1.7631	TG(64:13)	1022.8171	64	−0.3285
TG(51:2)	862.7858	51	0.6167	TG(54:4)	900.8015	54	0.5103	TG(64:3)	1042.9736	64	1.1823
TG(51:3)	860.7702	51	0.5211	TG(54:4)	883.7749	54	0.3293	TG(64:7)	1034.9110	64	−0.3104
TG(51:3)	860.7702	51	0.4740	TG(54:4)	900.8015	54	1.1544	TG(65:17)	1028.7702	65	−0.2053
TG(51:3)	860.7702	51	0.5139	TG(54:4)	900.8015	54	0.8247	TG(66:18)	1040.7702	66	−0.0511
TG(51:6)	854.7232	51	−0.3478	TG(54:5)	881.7593	54	0.7266	TG(66:7)	1062.9423	66	−0.2258
TG(52:0)	880.8328	52	0.5116	TG(54:5)	882.7909	54	−0.5341	TG(68:7)	1090.9736	68	−0.1900

**AD**											

TG(27:1)	528.4259	27	−0.4889	TG(52:2)	876.8015	52	1.2088	TG(55:6)	910.7858	55	0.2013
TG(32:0)	600.5198	32	−0.4528	TG(52:2)	876.8015	52	0.9103	TG(56:12)	895.6810	56	−0.5378
TG(36:0)	656.5824	36	−0.4128	TG(52:3)	874.7858	52	1.0580	TG(56:3)	930.8484	56	2.0573
TG(43:1)	752.6763	43	2.5381	TG(53:5)	884.7702	53	−0.4302	TG(56:5)	926.8171	56	−0.6091
TG(44:2)	764.6763	44	2.5937	TG(54:3)	902.8171	54	1.7832	TG(56:6)	924.8015	56	−0.5269
TG(44:5)	741.6028	44	2.5798	TG(54:3)	886.8222	54	−0.4955	TG(57:4)	942.8484	57	−0.6096
TG(50:1)	850.7858	50	−0.5152	TG(54:5)	882.7909	54	−0.4289	TG(60:3)	986.9110	60	2.8007

**Table 3:** Identification of potential lipid biomarkers.

Lipid	Symbol	CalcMz	Formula	Fatty acid	Fold change*
Triglyceride	TG	824.7702	C51 H102 O6 N1	16:0/16:0/16:0	1.69
Diglyceride	DG	696.6501	C43 H86 O5 N1	18:1/22:0	2.08
Phosphatidylcholine	PC	734.5694	C40 H81 O8 N1 P1	32:0	0.49
Phosphatidylserine	PS	850.5604	C47 H81 O10 N1 P1	41:5	0.68
Sphingomyelin	SM	725.5592	C41 H78 O6 N2 P1	36:4	1.17

Lipid molecules that were significantly altered in behavioral variant frontotemporal dementia (bvFTD) compared to both Alzheimer’s disease and control. CalcMz, calculated mass; fatty acid shows the number of carbon atoms and double bonds in fatty acid chains; fold change indicates the fold difference between bvFTD and control; *p < 0.005.

### Analysis of Phospholipids

**Figure 3:**
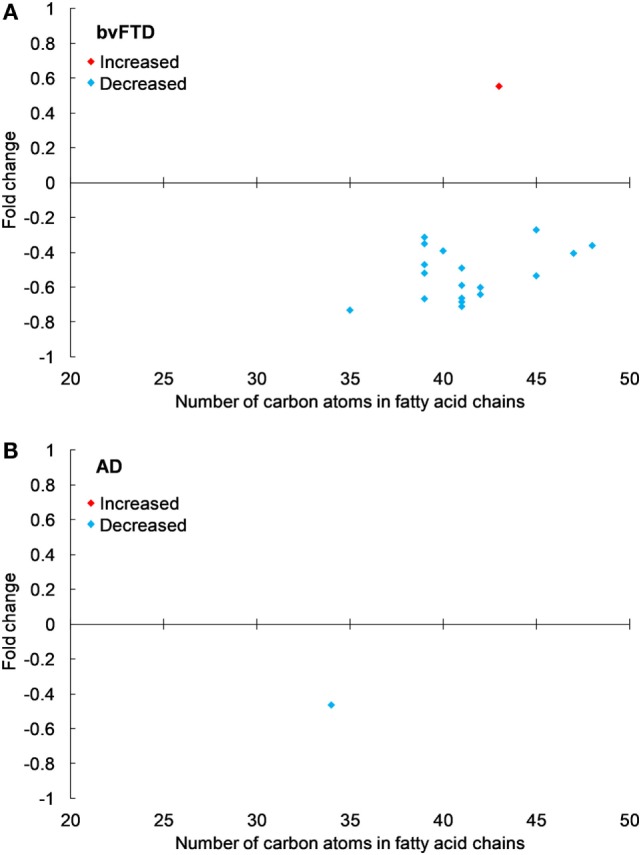
Phosphatidylserine species that were significantly altered in **(A)** behavioral variant frontotemporal dementia (bvFTD) and **(B)** Alzheimer’s disease (AD) compared to control. Each symbol represents a lipid species with different number of carbon atoms in fatty acid chains; red symbols are those that are significantly increased and blue symbols significantly decreased; *p* < 0.05. The data presented are fold changes on linear scale. Zero represents no change; 0.5 fold change represents 50% increase; and −0.5 represents 50% decrease.

The next class of lipids we investigated was phospholipids. Phospholipids are a major component of all cell membranes. In blood plasma, phospholipids are a major component of all lipoprotein membranes. A typical phospholipid molecule consists of a hydrophilic phosphate head and two lipophilic (hydrophobic) fatty acid chains. Their amphiphilic (both hydrophilic and lipophilic) property allows them to form lipid bilayers in the case of cell membranes and lipid monolayers in the case of lipoprotein membranes. The phosphate group is usually modified with simple organic molecules giving rise to different subclasses. The six major subclasses are PC, PS, PE, PI, PG, and PA. An analysis of total abundance of this class showed that PC was by far the most abundant subclass, followed by PS, PE, PG, PA, and PI (Table 1). There was a significant decrease in PS compared to both control and AD (Figure 1). PG was also significantly decreased in bvFTD compared to control only (Figure 1). There were no significant changes in any of other subclasses with a non-significant decrease (*p* = 0.092) in PC in bvFTD compared to control (Table 1). We also analyzed the level of individual species and have found that 18 PS species were significantly decreased in bvFTD (Figure 3A; Table 4). In contrast, only one PS species was significantly decreased in AD (Figure 3B; Table 4). None of the species overlapped between bvFTD and AD. Likewise, five PG species were significantly decreased in bvFTD and only one was significantly decreased in AD. Similarly, 87 PC species were significantly decreased in bvFTD and only 11 were significantly decreased in AD. We have identified one PS and one PC species that could be potentially used for developing biomarkers for bvFTD (Table 3).

**Table 4:** Individual phosphatidylserine (PS) species that were significantly altered (*p* < 0.05) in behavioral variant frontotemporal dementia (bvFTD) and Alzheimer’s disease (AD) compared to control.

Species	CalcMz	C atoms	Fold change	Species	CalcMz	C atoms	Fold change	Species	CalcMz	C atoms	Fold change
**bvFTD**											

PS(35:1)	774.5291	35	−0.7322	PS(41:4)	852.5760	41	−0.6634	PS(43:4)	880.6073	43	0.5540
PS(39:3)	826.5604	39	−0.3133	PS(41:5)	850.5604	41	−0.7109	PS(45:10)	896.5447	45	−0.2712
PS(39:4)	824.5447	39	−0.5198	PS(41:5)	850.5604	41	−0.6849	PS(45:6)	904.6073	45	−0.5349
PS(39:4)	824.5447	39	−0.3506	PS(41:6)	848.5447	41	−0.4904	PS(47:11)	922.5604	47	−0.4057
PS(39:5)	822.5291	39	−0.4708	PS(41:6)	848.5447	41	−0.5897	PS(48:10)	938.5917	48	−0.3612
PS(39:5)	822.5291	39	−0.6669	PS(42:6)	848.5811	42	−0.6424				
PS(40:5)	822.5654	40	−0.3915	PS(42:6)	848.5811	42	−0.6019				

**AD**											

PS(34:0)	762.5291	34	−0.4638								

### Analysis of Sphingolipids

Sphingolipids (also called glycosylceramides) are a class of lipids containing a backbone of So. A basic form of sphingolipid is Cer, which is a precursor to many other sphingolipids, including glucosylceramide and SM. They are a major component of cell membranes and play a role in cell signaling. In blood plasma, they are associated with lipoproteins. The major subclasses identified in our lipidomics analysis were Cer, monoglycosylceramide (CerG1), ceramide phosphate (CerP), SM, So, and sphingosine phosphate (SoP). There were no significant changes in the total abundance of any of the sphingolipid subclasses in bvFTD compared to control (Table 1). However, we have identified one SM species that was significantly increased in bvFTD compared to control and AD. This species could potentially serve as a biomarker for bvFTD (Table 3).

### Analysis of Sterols

Cholesterol is the most abundant sterol in plasma. Two sterols were identified in our lipidomics analysis, ChE and zymosteryl ester (ZyE). ChE is composed of a cholesterol molecule with a single fatty acid chain. ChE is the storage form of cholesterol, and in blood plasma, the majority of cholesterol transported by lipoproteins are in this form. ZyE is derived from zymosterol, which is an intermediate in cholesterol biosynthesis. In terms of total abundance, there were no significant changes in either ChE or ZyE in bvFTD compared to control (Table 1). We have identified 44 ChE and 22 ZyE species in this lipid class (Table 1); however, none of them were significantly altered (i.e., *p* < 0.005) to be considered as a potential biomarker.

### Analysis of Dietary (Plant) Lipids

In our lipidomics analysis, we have identified a number of dietary lipids. These were monogalactosyldiacylglycerol (MGDG), stigmasteryl ester (StE), sitosteryl ester (SiE), and wax ester (WE). These lipids are normally derived from food matter originating from plants or marine creatures. The plant lipids (MGDG, StE, and SiE) are not synthesized in humans but are derived entirely from diet. MGDG is a component of the thylakoid membrane of plant chloroplasts and as such it is one of the most abundant lipids in nature. It is consumed by humans as a part of vegetable diet. SiE is a major dietary plant sterol (also called phytosterol). It is found in vegetable oils, nuts, and vegetables. StE is also a phytosterol and is found in vegetables, legumes, nuts, seeds, and a number of medicinal herbs. WE is commonly found in arthropods (e.g., shrimps), shellfish, certain fish species, as well as in whole grain cereals, seeds, and nuts. The total abundance of MGDG was significantly decreased in bvFTD compared to controls and AD (Figure 1). Likewise, the total abundance of SiE was significantly decreased in bvFTD compared to controls (Figure 1). SiE was also significantly decreased in AD compared to control (Figure 1). The total abundances of StE and WE were unaltered in both bvFTD and AD (Table 1).

### Correlations

Pearson correlation was used to test for any association between lipids. TG was positively correlated with its two precursors DG (*r* = 0.844, *p* = 3.8 × 10^−15^) and MG (*r* = 0.611, *p* = 1 × 10^−6^) as expected, positively validating our lipidomics data. TG was negatively correlated with ChE (*r* = −0.405, *p* = 0.003) and ZyE (*r* = −0.501, *p* = 0.0002). All six subclasses of the phospholipid class correlated strongly with each other (all six). For example, the correlation between PC and PE was *r* = 0.783, *p* = 6.8 × 10^−12^, and between PS and PI was *r* = 0.719, *p* = 1.9 × 10^−9^. The correlation among phospholipid subclasses is expected to be positive since their distribution in lipoprotein membranes is normally constant. All phospholipids correlated positively with sphingolipids Cer, SM, and So, which are also components of lipoprotein membranes. PC was the only phospholipid that correlated with ChE (*r* = 0.441, *p* = 0.001). All sphingolipids correlated positively with each other as expected. For example, the correlation between Cer and SM was *r* = 0.654, *p* = 1.5 × 10^−7^, and between SM and So was *r* = 0.685, *p* = 2.2 × 10^−8^. SM was positively correlated with ChE (*r* = 0.516, *p* = 9.1 × 10^−5^). ChE was strongly correlated with its precursor ZyE (*r* = 0.813, *p* = 2.5 × 10^−13^) as expected, once again positively validating our lipidomics data. Of interest, the plant lipid MGDG correlated positively with all the phospholipids, in particular PS (*r* = 0.669, *p* = 5.9 × 10^−8^) and PI (*r* = 0.697, *p* = 9.7 × 10^−9^). Also of interest, all of the esters (ChE, ZyE, StE, SiE, and WE) correlated positively with each other. For example, the correlation between ChE and WE was *r* = 0.844, *p* = 3.9 × 10^−15^.

## Discussion

Lipidomics has been increasingly used to study lipid dysfunction and has provided invaluable data in understanding disease pathogenesis of a number of diseases. One clear and consistent finding from all studies is the vast array and complexity of lipid species present in human plasma. This has provided the scope to utilize lipidomics in disease classification and biomarker development. As of present, lipidomics of bvFTD has not been reported. Our study therefore represents the first lipidomics analysis of blood samples of patients with bvFTD. Our aim was to establish a differentiating plasma lipid profile for bvFTD and to uncover changes in lipid levels associated with this disease. Identification of altered lipid species in bvFTD would allow development of a potential objective biomarker for bvFTD. Furthermore, analysis of lipid changes in bvFTD would facilitate our understanding of the metabolic changes that are evident in bvFTD patients and of the lipid loss that is directly associated with neurodegeneration.

The first class of lipids we analyzed was glycerolipids; subclasses TG, DG, and MG. Emerging evidence indicates that hypertriglyceridemia is a characteristic of bvFTD, and a recent measurement of TG in bvFTD (by an indirect enzymatic method) showed that it was significantly increased (based on our statistical analysis) compared to controls (18). Consistent with this, we have shown that TG was significantly increased in bvFTD. There are a number of factors that can contribute toward hypertriglyceridemia and these include uncontrolled diabetes mellitus, obesity, sedentary lifestyle, and genetic determinants. We know that bvFTD patients have eating disorder, i.e., binge eating and increased intake of sweet foods (15, 22), and this is likely to have contributed toward hypertriglyceridemia. This is supported by the fact that bvFTD patients have significantly higher blood insulin levels compared to healthy controls (23). Elevated TG levels, as well as DG and MG levels, and elevated insulin levels suggest a state of insulin resistance. Diets high in refined carbohydrates (sugar) are known to increase TG levels. This correlation is stronger for those with high BMI (≥ 28) (24). The average BMI for bvFTD is 29.65 (17), and therefore, bvFTD patients have a higher risk of developing carbohydrate-induced hypertriglyceridemia. It is interesting to note that heavy alcohol consumption can also induce hypertriglyceridemia (25, 26); increased alcohol consumption is also a characteristic of bvFTD. Unabated hypertriglyceridemia, accompanied by low HDL-cholesterol, would increase the risk of coronary heart disease. There is also evidence that hypertriglyceridemia with low HDL-cholesterol is associated with an increased risk of developing mild cognitive impairment (27, 28). Further research is required to understand the relationship between hypertriglyceridemia, bvFTD, and cognitive impairment.

Previous lipidomics analyses (not in bvFTD) showed that a considerable number of TG and DG species were closely associated with dyslipidemia, and TG 50:2, 52:2, 52:3, and 52:4 were the most abundant species (29). In a twin study, consisting of 14 pairs of young-adult monozygotic twins discordant for obesity, several TG species correlated significantly with BMI and subcutaneous fat measurement (30). A prominent TG that was identified from the study was TG 56:4 with the suggestion that this species may serve as a potential biomarker for the detection of acquired obesity. In our lipidomics analysis, TG 56:4 was the second most significantly increased TG species with a *p* value of 0.0006.

Phospholipids are abundant in blood plasma as a major component of lipoprotein membranes. We have identified six subclasses of phospholipids. Our data showed significant decreases (based on our statistical analysis) in the total abundance of PS and PG in bvFTD compared to control and an overall non-significant trend for a decrease in other phospholipids in bvFTD. The total abundance of PS was also significantly decreased in bvFTD compared to AD; PS was the only lipid subclass in all non-plant lipid subclasses measured that was significantly altered in bvFTD compared to both control and AD. A body of evidence suggests that decreases in PS level in the brain could be associated with cognitive decline (31, 32). As a major component of neuronal membrane and myelin, PS is essential for their formation and maintenance (33, 34). Studies with old rats have shown that oral intake of PS increased neurotransmission signals (35–37) and improved learning and memory (38–40). PS is absorbed into the bloodstream following ingestion and readily crosses the blood–brain barrier (41, 42).

A comparison of individual species showed numerous PS, PG, and PC species that were significantly decreased in bvFTD. In contrast, very few species were altered in AD. PC is the most abundant phospholipid in all lipoproteins, especially in HDL. As expected, it was by far the most abundant phospholipid in our plasma samples. Furthermore, the PC subclass contained by far the most number of putative species (i.e., 1,062). Its abundance and structural diversity suggest critical involvement in physiological processes. In fact, PC has an important and unique function, in that it is the only phospholipid which is currently known to be required for lipoprotein assembly and secretion (43). An interesting observation from our study was that reduced levels of phospholipids in bvFTD (indicating reduced numbers of HDL) corroborates the previous finding that HDL-cholesterol is reduced in bvFTD (18). In support, there was a positive correlation between PC and ChE. These results when put together suggest an increased risk of hypoalphalipoproteinemia in bvFTD.

The levels of sphingolipids were largely unaltered in bvFTD and AD cohorts. A previous study reported elevated plasma levels in AD, although these changes were observed in early AD or mild cognitive impairment (44). Earlier lipidomics analyses of healthy controls have identified >200 sphingolipid species (45, 46); we have identified 329 sphingolipids species with 229 SM species alone. The overall levels of ChE (as well as ZyE) were unaltered in bvFTD or AD. This result was in accordance with a previous study, in which total cholesterol (measured by an enzymatic colorimetric assay), was unaltered in bvFTD (18).

In our lipidomics analysis, we also identified three groups of dietary lipids (MGDG, StE, and SiE) originating from plants. These lipids are not synthesized in humans but are derived entirely from diet, and therefore, they can be used as indicators of what food people ate. Both MGDG and SiE were significantly decreased in bvFTD (both unchanged in AD). This suggests that the patients with bvFTD consume reduced amounts of plant matter (i.e., vegetables and fruits) compared to controls. Another plant sterol, StE, was unaltered in bvFTD. The level of WE (e.g., shrimps and shellfish) was also unaltered in bvFTD. Therefore, the consumption of only certain food categories is altered in bvFTD.

A number of protocols/strategies have been developed for lipidomics of human plasma (47), and we have used one of the most common and efficient strategies—a single phase extraction method followed by liquid chromatography, ESI, and tandem mass spectrometry. This strategy allows the accurate measurement of literally hundreds, if not thousands, of different lipid species in a single biological sample. A watershed for lipidomics has been the development of ESI in mass spectrometry. ESI is a technique to generate ions from molecules. The advantage of ESI over other techniques is that very little fragmentation of molecules occurs during the ionization process, although this is still a limitation. The structural diversity is particularly apparent with TG and PC, for which we have identified 1,115 and 1,062 distinct molecular species, respectively. Recent improvements in mass spectrometry in lipids have allowed detection and quantification of individual molecular species. To determine the efficiency of recovery of lipids, our plasma samples were spiked with a number of ISTDs of known quantity. All ISTDs achieved expected high recovery rates, i.e., >98%. This indicated that the extraction method, based on the method of Bligh and Dyer (20), was thorough and resulted in a comprehensive recovery of lipids. Our choice for this extraction method was based on a previous study, in which five different extraction methods were tested for blood plasma (48). The Bligh and Dyer method was considered the best in terms of coverage and reproducibility across a variety of lipid classes, i.e., “broad-based” lipidomics. Nevertheless, limitations still exist because not all (100%) of lipids present in a given sample can be analyzed. Further limitations lie in the statistical approach we used to analyze the lipidomics dataset. An empirical Bayesian approach is an alternative that would increase power and reduce false positives.

Like transcriptomics and proteomics, lipidomics gives the opportunity to investigate biological changes at a systems level to allow the identification and measurement of distinct metabolites that change with different disease states. Limitations and questions still remain as to why there are so many species of lipids in blood and what are their functions. This conundrum is somewhat similar to the enormous stretches of non-coding DNA in the genome that was once called “junk DNA,” these are now known to play important roles in gene regulation and epigenetics.

We have demonstrated that significant lipid changes occur in the blood as a consequence of bvFTD. We have identified a number of individual lipid species from each lipid class that could potentially be used for developing biomarkers for bvFTD. Future work would involve developing a combination of lipid species that could serve as a reproducible objective lipid signature to differentiate bvFTD from AD and controls, in particular at preclinical and various stages of disease progression. Our lipidomics analysis has not only identified lipids that change in bvFTD, it has also provided data that could be utilized to identify biosynthetic enzymes and metabolic pathways that are altered in bvFTD. In conclusion, our study has provided new insights into an unrecognized perturbed pathology in bvFTD, providing evidence in support of considerable lipid dysregulation in bvFTD.

## Ethics Statement

This study was approved by the University of New South Wales human ethics committee (approval number: HC12573), and the blood was obtained following written informed consent from the participant and/or primary carer.

## Author Contributions

WK and GH were involved in the conception and design of study, and all authors (WK, EJ, RP, YH, RA, OP, JH, and GH) participated in the acquisition and analysis of data and in the drafting of manuscript.

## Conflict of Interest Statement

The authors declare that the research was conducted in the absence of any commercial or financial relationships that could be construed as a potential conflict of interest.
